# Stability of the effects of a social competence training program for children with oppositional defiant disorder/conduct disorder: a 10-month follow-up

**DOI:** 10.1007/s00787-021-01932-1

**Published:** 2022-03-13

**Authors:** Teresa Del Giudice, Timo Lindenschmidt, Martin Hellmich, Christopher Hautmann, Manfred Döpfner, Anja Görtz-Dorten

**Affiliations:** 1 Faculty of Medicine, School of Child and Adolescent Cognitive Behavior Therapy (AKiP), University Hospital Cologne, University of Cologne, Cologne, Germany; 2 Faculty of Medicine, Department of Child and Adolescent Psychiatry, Psychosomatics and Psychotherapy, University Hospital Cologne, University of Cologne, Cologne, Germany; 3 Faculty of Medicine, Institute of Medical Statistics and Computational Biology, University Hospital Cologne, University of Cologne, Cologne, Germany

**Keywords:** Oppositional defiant disorder, Conduct disorder, Cognitive behavioral therapy, Long-term effects

## Abstract

The stability and effectiveness of the Treatment Program for Children with Aggressive Behavior (THAV) in terms of reducing behavioral problems in children with oppositional defiant disorder (ODD) and conduct disorder (CD) were examined at a 10-month follow-up (FU). A total of 76 families and their children (boys aged 6–12 years), who previously participated in a randomized controlled trial comparing THAV with an active control group, took part in the 10-month FU assessment. Outcome measures were rated by parents and included the evaluation of child aggressive behavior, prosocial behavior, problem-maintaining and problem-moderating factors, and comorbid symptoms. Linear mixed models for repeated measures (MMRM) were conducted. The results revealed that THAV effects remained stable (problem-maintaining and problem-moderating factors; comorbid symptoms) and even partially improved (aggressive behavior; ADHD symptoms) over the FU period. Additionally, the differences between the THAV intervention group and the control group, which were apparent at the end of the treatment (post), mainly also remained at the FU assessment. It can be concluded that THAV is an effective and stable intervention for boys aged 6–12 years with ODD/CD.

## Introduction

Oppositional defiant disorder (ODD) and conduct disorder (CD) are among the most common disorders for which children and adolescents are referred for mental health treatment [1]. Longitudinal studies have shown that ODD/CD are highly stable over time and predict later emotional and behavioral problems in adolescence and adulthood [2–4]. With regard to psychotherapeutic interventions, ODD/CD are notably responsive to parent management training (PMT) [5–8], child-centered treatments (cognitive behavioral interventions (CBT) including social skills training) [9, 10], and a combination of PMT and CBT programs [5, 11–13].

While PMT is considered as the gold standard, this approach still has some limitations, and some factors remain untreated, in particular those related to the child (e.g., child’s inability to handle anger due to affect regulation deficiencies) [14]. Moreover, regarding therapeutic effects on behavioral problems outside the family (e.g., behavior at school; peer relationship problems), CBT interventions that focus on social skills and cognitive interventions (e.g., problem-solving and anger management strategies) seem to increase the effects of PMT [13, 15, 16].

However, many studies only consider the post-treatment effects and neglect to examine the long-term effects, which are of great importance given the negative long-term prognoses of ODD/CD. In this context, a meta-analysis by van Aar and colleagues [17] encompassing 40 studies examined the long-term effects [follow-up (FU) assessment up to 3 years] of PMT on disruptive child behavior and found that the post-treatment effects remained stable. However, the long-term effects of CBT interventions were not considered. Furthermore, a meta-analytic review by Fossum and colleagues [18] identified 56 studies (published between 1984 and 2010) that examined the longer term impact (with a mean FU period of 8.9 months; SD = 6.5) of PMT (*k* = 34), CBT (*k* = 7), a combination of PMT and CBT (*k* = 12) or family therapy (FT; *k* = 3) on conduct problems and oppositional behaviors (within the clinical range before treatment). Overall, the findings again indicated a stability of treatment effects. None of the included studies found a significant deterioration in ODD/CD symptoms in the FU period compared to post-treatment, and overall, there was even a small (non-significant) further reduction in conduct problems after treatment was over (effect size = 0.08). Moreover, the meta-analysis revealed that changes in conduct problems were significantly larger in the studies that examined PMT in combination with CBT or CBT alone compared to studies that examined PMT alone or FT. The study by Lochman and Wells [13] comes to comparable findings. Their analyses showed that the Coping Power Program (a child CBT program) have sustained effects 1 year after the program has ended, especially when parent components are added. Children of the intervention group showed lower rates of self-reported covert delinquent behavior and of parent-rated substance use at FU that at the baseline than did the control group.

However, most of the studies considered here predominantly examined the stability of treatment effects (i.e., within-group change: comparison of post-treatment versus FU), while only a small number of studies, e.g., [19, 20], also investigated whether there were differences between the treatment and control group at FU (i.e., between-group comparison). These studies mostly did not find any significant differences between the groups at FU, possibly due to the fact that control group participants had received adequate therapy in the interim.

Overall, it appears that the number of studies on long-term effects of cognitive interventions in ODD/CD is considerably low. A closer look also shows that there are hardly any studies that analyze the difference between the treatment and the control group at FU. Further studies are strongly needed in this area.

The aim of the present study was therefore to evaluate the long-term effects of the Treatment Program for Children with Aggressive Behavior (THAV) [21]. THAV mainly consists of a patient-focused intervention program (CBT), which is combined with parent interventions (PMT) and teacher- and peer-focused interventions according to the individual needs of the patient.

The effectiveness of the program has been demonstrated in two previous analyses [11, 22]. In a study employing a within-subject design which included 6–12-year-old boys with peer-related aggressive behavior, growth rates for all parent-rated outcome measures were found to be significant during treatment, and a comparison with the waiting phase indicated a stronger decrease in aggressive behavior and a stronger increase in prosocial behavior during treatment [22]. In a randomized controlled trial (RCT) evaluation, THAV (*n* = 50) was compared with an active control involving group play (PLAY; *n* = 41). The between-group evaluation showed mostly moderate treatment effects for THAV compared to PLAY in parent ratings on aggressive behavior, comorbid symptoms, psychosocial impairment, quality of life, parental stress, and negative expressed emotions at post-assessment [11].

In the current analysis, we subsequently examined whether these effects (based on parent ratings) remain at a 10-month FU assessment. Besides testing the stability of treatment effects, the present study also seeks to shed light on the differences between THAV and PLAY at 10-month FU. In sum, our hypotheses were as follows: (a) improvements in child behavior (based on multiple parent-rated outcome measures) through THAV treatment would be sustained 10 months later (comparison of post-treatment versus 10-month FU controlling for pre-treatment); (b) the significant effects of THAV compared to PLAY shown at post-assessment would also be apparent at 10-month FU.

## Methods

### Ethical considerations

The protocol of the study conducted at the University of Cologne (Clinical trials.gov Identifier: NCT01406067) was approved by the ethics committee of the University Hospital, Cologne. The study was performed in accordance with the ethical standards laid down in the 1964 Declaration of Helsinki and its later amendments. All participants provided written informed consent.

### Participants and procedure of the study

**Fig. 1:**
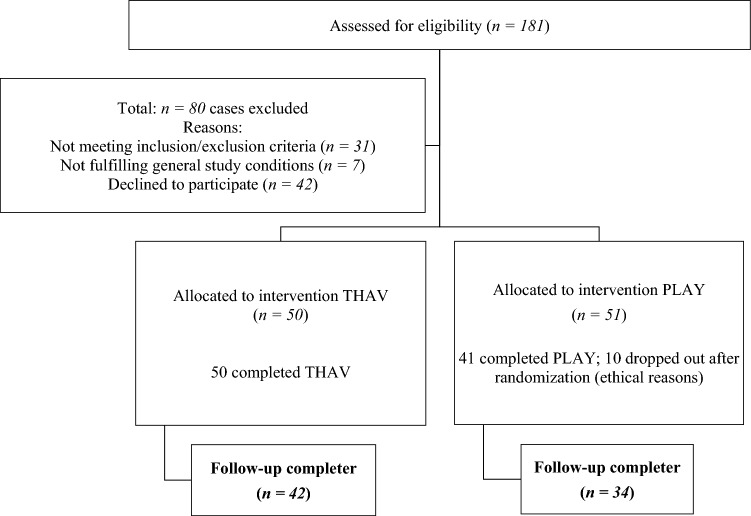
Participant flow diagram

Figure 1 describes the participant flow. For pre-assessment, a total of 181 patients were referred to the outpatient unit of the clinic between January 2011 and January 2013 due to severe conflicts with peers and/or family members. Based on inclusion and exclusion criteria, they were screened for eligibility. Children were eligible for inclusion if they were male, aged 6–12 years, with an IQ > 80 (in the Culture Fair Intelligence Test) and an ICD-10 diagnosis of CD (F91), mixed disorder of conduct and emotions (F92), or hyperkinetic conduct disorder (F90.1), as determined using the semi-structured interview for disruptive behavior disorders (ODD, CD; DCL-DBD) of the German Diagnostic System for Children and Adolescents (DISYPS-II) [23]. Moreover, since the goal of the intervention was to treat peer conflicts, peer-related aggressive behavior had to cause persistent impairments in relationships with other children (based on clinical rating in the semi-structured interview). In addition, the child had to have a high symptom score (Stanine score ≥ 7) on the German Symptom Checklist for Disruptive Behavior Disorder (SCL-DBD) total score of the DISYPS-II [23] at the pre-assessment. Exclusion criteria were the presence of a primary comorbid disorder (e.g., autism) according to the judgment of the clinician, a planned change in medication in a child receiving psychotropic medication, other child psychotherapy, and insufficient German-language skills of the parents.

Of the original sample of 181 patients, 38 patients were excluded, because they did not fulfill the inclusion criteria or because they met exclusion criteria (e.g., other active child psychotherapy). Further 42 patients were not interested in participating in the study. Thus, a total of 101 patients were randomized to THAV or PLAY, using a block randomization with a block size of 4 and random selections from all 6 permutations. Ten patients dropped out from the PLAY condition after randomization due to ethical objections of the therapists (i.e., that active THAV treatment was necessary, because the child was at risk of school exclusion due to behavioral problems). Except for these 10 patients, all other randomized patients completed the THAV (*n* = 50) and PLAY (*n* = 41) conditions. We decided to exclude these patients from further analysis, because an imputation of missing values in this control group (e.g., with last observation carried forward) would have favored the treatment group. These ten patients which are excluded from PLAY did not differ statistically on the SCL-DBD symptom score (*t* = 1,20, *df* = 49, *p* = 0.237) at pre-2. Besides these 10 patients, all other randomized patients completed the THAV and PLAY conditions.

After 10 months, the participating families were reassessed, and were then asked to participate in the FU assignment. The percentage of participants available at 10-month FU was approximately 84% (THAV: *n* = 42; PLAY: *n* = 34) of the original sample. The reduced number of participants was mainly due to the fact that some families no longer wished to participate in the study or had moved away and could no longer be contacted.

### Measurement time points

The dependent measures were administered to both groups at five time points: Pre 1 (before a waiting period; patients were assessed for eligibility at the Screening), Pre 2 (after the baseline waiting period of 6 weeks; patients were reassessed and randomized), Within (after half of treatment had been completed), Post (after a 24-week treatment period; patients were reassessed), and FU (10 months after post; patients were reassessed). In the present paper, the data of Pre 2, Post, and FU (controlling for pre-treatment) are analyzed.

### Interventions

The treatment and control interventions were conducted by 13 experienced child therapists or therapists in training. The same therapists administered both the treatment and control interventions. The therapists received weekly group supervision by a senior child therapist (A.G-D). With regard to treatment integrity and treatment adherence, therapists indicated that they spent 88% of the total treatment time on specific modules of the THAV treatment program. For the PLAY condition, only the general outlines of the intervention were given, indicating which interventions were/were not allowed. Therapists were supervised regularly, and treatment integrity was rated globally as good-to-excellent by the supervisor. In the following section, THAV and PLAY are described in detail. A comparative overview can also be found in Table 1.

**Table 1:** Description of treatment and control interventions

	THAV	PLAY
Type of treatment	CBT intervention	Educational group play (3–5 children)
Number of sessions	24 weekly child sessions (each 45 min)	12 fortnightly sessions (each 90 min)
Individualized vs. standardized		
	Individually tailored to address the need of child and his/her parents	Not tailored to specific problems of the child and the parents
Main component	Patient-focused interventions: (1) psychoeducation and development of a therapeutic relationship; (2) social-cognitive interventions; (3) anger control training; (4) social problem-solving and skills training; and (5) relapse prevention	Patient-focused interventions: (1) resource-activating exercises; (2) practicing prosocial interactions in groups; (3) conflict resolution guidance
No implementation of specific problem-solving techniques or other cognitive interventions		
Number of parent sessions	An average of 8 sessions or shorter contacts	Two parent group sessions (90 min)
Content of parent sessions	(1) Identifying problems and competencies; (2) Teaching parents how to device social rules, communicate effective commands; (3) Teaching how to use rewarding the child, how to use appropriate methods of punishment; (4) Identify and modify parental dysfunctional thoughts	psychoeducation on appropriate general parenting strategies
Additional modules	Involvement of teachers if needed	–

Notes. *THAV* treatment program for children with aggressive behavior, *PLAY* group play control

### The treatment program for children with aggressive behavior (THAV)

THAV is a CBT intervention for children aged 6–12 years with peer-related overt aggressive behavior. It provides individualized treatment for problem-maintaining factors in specific daily life situations, which each respective child has experienced in the previous weeks. Depending on the problem-maintaining factors specific to each individual, THAV aims to modify social-cognitive information processing, impulse control, social problem-solving, social skills, and social interactions in these situations. It combines patient-, parent-, teacher-, and peer-focused interventions. Patient-focused interventions are the main component, while parent-, teacher-, or peer-focused interventions are added according to the individual needs of the patient. Treatment with THAV comprises 24 weekly child sessions (lasting for 45 min each) and additional sessions or shorter contacts with parents. According to the study inclusion criteria, all patients had high symptom scores (Stanine scores ≥ 7) prior to treatment on the parent-rated SCL-DBD total score. At post-assessment, 66% of patients in the THAV condition had dropped below this cut-off (indicating normalization). For a more detailed description, see Görtz-Dorten and colleagues [11, 22].

### Group play (PLAY)

The active control condition (PLAY) consisted of educational group play, with 3–5 children in each group. Each group received 12 fortnightly sessions (lasting for 90 min each) over 24 weeks. The sessions utilized techniques to activate resources and provided the opportunity to practice prosocial interactions in groups. For instance, social play interactions and projects were offered that aimed to develop cooperative interaction (e.g., making a movie together, constructing game materials together and then playing with them) or to provide the opportunity to practice socially competent ways of solving conflicts (e.g., sharing, negotiating) [24]. Children were supported to solve conflicts and to develop cooperative interactions, and were also praised for socially competent behavior and for their own general competencies (e.g., being good at sports). No specific problem-solving techniques (e.g., development of alternative solutions, evaluation of solutions) or other cognitive interventions (e.g., identification of anger thought) were implemented. Moreover, these sessions did not provide skills training with role-play and rehearsal, or interventions to support transfer to real life (e.g., therapeutic homework assignments). Parents attended two parent group sessions (90 min each), during which they received psychoeducation on appropriate general parenting strategies. However, these general parenting strategies were not tailored to the specific problems of the child and the parents were not trained to implement these techniques in their daily parenting behavior. At post-assessment, 26% of patients in the PLAY condition dropped below the cut-off on the parent-rated SCL-DBD total score. A Chi-square test of the distribution of deteriorated or unchanged patients compared to improved patients showed a statistically higher percentage of improved patients in the THAV condition compared to the PLAY condition. For a more detailed description, see Görtz-Dorten and colleagues [11].

### Assessment

*The Questionnaire for Aggressive Behavior of Children* (FAVK: Fragebogen zum aggressiven Verhalten von Kindern) [25] is a parent-rating scale which assesses the following maintaining factors of peer- and adult-related aggression: (1) disturbance of social-cognitive information processing, (2) disturbance of social skills, (3) disturbance of impulse control, and (4) disturbance of social interaction. Parents rated each of the 25 items on a 4-point Likert scale ranging from 0 (“not at all”) to 3 (“very much”), with higher scores indicating a higher degree of symptoms. For total scores on peer-related aggression-maintaining factors (FAVK-total *peer*) and adult-related aggression-maintaining factors (FAVK-total *adult*), mean standardized scores (sum of item scores)/(number of items) were calculated. Confirmatory factor analyses (CFA) of the parent ratings confirmed the hypothetical four-factor model, and satisfactory internal consistencies were found (Cronbach’s α > 0.70) [26].

*The SCL-DBD* [23] is part of the DISYPS-II [23] and measures symptoms of ODD and CD according to the DSM-IV and ICD-10 as well as prosocial behavior. The questionnaire comprises 25 items that are rated with regard to their severity on a 4-point Likert scale ranging from 0 (“not at all”) to 3 (“very much”), with higher scores indicating a higher degree of symptoms/prosocial behavior. By averaging the associated item scores, three subscale scores (oppositional behavior, antisocial behavior, and prosocial behavior) and a total score can be formed. The SCL-DBD has been shown to be a factorially valid and internally consistent (Cronbach’s α = 0.69–0.90) parent and teacher questionnaire [27]. When diagnostic accuracy was examined using receiver operating characteristic analyses, the SCL-DBD was found to be excellent at discriminating between children with ODD/CD in a community sample and those in a clinical sample (area under the curve [AUC] = 0.91), and showed satisfactory diagnostic accuracy for detecting ODD/CD within the clinical sample (AUC = 0.76) [27].

*The Child Behavior Checklist for Ages 4–18* (CBCL 4–18) [28] is a parent-rated questionnaire assessing a broad spectrum of child behavioral and emotional problems. It consists of 118 problem behavior questions associated with two superordinate scales that reflect externalizing and internalizing syndromes, with higher scores indicating a higher degree of symptoms. Furthermore, the items can be aggregated into eight syndrome scales; however, we did not calculate these scales in the present study. The German version of the CBCL has been shown to be robust and highly reliable (CBCL: Cronbach’s α = 0.69–0.93), and all subscale scores as well as the total score have shown factorial validity [29].

*The parent-rated Symptom Checklist for attention deficit hyperactivity disorder* (SCL-ADHD) from the DISYPS-II [23] was used to assess all ADHD symptoms according to ICD-10 and DSM-IV criteria, as well as competencies (endurance, attention, and reflexivity). Parents rated each of the 20 items on a 4-point Likert scale ranging from 0 (not at all) to 3 (very much), with higher scores indicating a higher degree of symptoms. The SCL-ADHD has been shown to be a factorially valid and internally consistent (Cronbach’s α = 0.80–0.94) parent questionnaire [30, 31].

### Statistical analysis

Differences between THAV and PLAY regarding the support received during the FU period (psychological outpatient treatment, pharmacotherapy, inpatient treatment, and training therapy), which was assessed by questionnaire (parent rating), were evaluated by Chi-square test. The stability of effects (comparison of post-treatment versus 10-month FU controlling for pre-treatment), and the differences between THAV and PLAY at 10-month FU, were evaluated using linear mixed models for repeated measures (MMRM) with the fixed effects group, time, baseline value, and the interaction group*time (REML, Satterthwaite adjustment for small sample size). Thus, outcome values are implicitly handled under the assumption “missing at random”: [32]. A random cluster effect was specified by group (to account for partial clustering), and for the residuals, an unstructured covariance matrix was allowed over time. Specific contrasts with 95% confidence intervals and *p* values were derived from estimated marginal means. Effect sizes (Cohen's d) were calculated, again with 95% confidence intervals. Calculations were performed with Stata/SE 16.1 [33] and SPSS Statistics 26 [34].

Furthermore, Jacobson’s Reliable Change Index (RCI) was calculated [35] to indicate clinically significant change. Based on means of the SCL-DBD *total* (pre-treatment vs. FU), patients were divided into three groups: (1) worsened, (2) unchanged, and (3) improved.

## Results

Sample characteristics including ICD-10 diagnosis at baseline, age at FU assessment, treatment received during the FU period, and average waiting time are reported in Table 2. Chi-square tests indicated no significant differences between THAV and PLAY regarding treatment received during the FU period [psychological outpatient treatment (*Χ*^*2*^(1) = 3.25, *p* = 0.07); pharmacotherapy (*Χ*^*2*^(1) = 0.03, *p* = 0.95); training therapy (e.g., on reading and spelling training) (*Χ*^*2*^(1) = 0.74, *p* = 0.39)].

**Table 2:** Sample characteristics at follow-up assessment

Variable	Total sample (*N* = 76)	THAV (*n* = 42)	PLAY (*n* = 34)
ICD-10 diagnosis at baseline			
F91.1	2 (2.6%)	1 (2.4%)	1 (2.9%)
F91.2	1 (1.3%)	1 (2.4%)	0
F91.3	57 (75.0%)	25 (76.2%)	32 (73.5%)
F92.8	2 (2.6%)	1 (2.4%)	1 (2.9%)
F90.1	14 (18.4%)	7 (16.7%)	7 (20.6%)
Age (years): mean (SD) at FU	9.9 (1.86)	10 (1.89)	9.9 (1.86)
Treatment during FU period (yes: frequency)			
Psychological treatment	20	8	12
Medical therapy	12	7	5
Inpatient treatment	0	0	0
Training therapy	5	2	3
Waiting period: months (SD)	10.11 (2.96)	10.0 (3.04)	10.24 (2.88)

Notes: *THAV* Treatment program for children with aggressive behavior, *PLAY* group play control

Results of the MMRM including means and SDs (post and FU) as well as effect sizes are detailed in Table 3. On a descriptive level, both THAV and PLAY yielded a reduction in problem behavior from post-assessment to FU assessment, with effect sizes in the low-to-moderate range (d_post/FU_ = 0.06–0.43). These differences were not significant, with the exception of the scales SCL-DBD *ODD* for both THAV and PLAY, the SCL-ADHD *total* for THAV and the CBCL-*Internalizing problems* for PLAY. Here, the values at FU assessment were significantly lower than those at post-assessment.

**Table 3:** Patient outcomes (parent ratings) on main scales; means and standard deviations for the two assessment points, Cohen’s d effect sizes, and the results of the analysis of covariance (ANOVA)

Scale	Group	Descriptive statistics		MMRM			
		Mean (SD; *n*) Post	Mean (SD; *n*) FU	Contrast post-FU estimate (95% CI); *p* value	Effect size (gain)	Contrast PLAY-THAV (FU) Estimate (95% CI); p value	Effect size (Cohen’s d)
FAVK-total (peer)	THAV	0.91 (0.40; 48)	0.75 (0.54; 34)	0.12 (– 0.05 to 0.28); 0.16	0.30	0.30 (– 0.03 to 0.63); 0.07	0.47
	PLAY	1.16 (0.51; 35)	1.03 (0.62; 29)	0.11 (– 0.06 to 0.29); 0.21	0.22		
FAVK-total (adult)	THAV	0.63 (0.37; 48)	0.55 (0.39; 33)	0.09 (– 0.04 to 0.22); 0.17	0.24	0.16 (– 0.06 to 0.38); 0.15	0.37
	PLAY	0.83 (0.44; 35)	0.71 (0,50; 29)	0.10 (– 0.05 to 0.24); 0.18	0.23		
SCL-DBD (ODD)	THAV	1.03 (0.53; 48)	0.80 (0.53; 40)	0.23 (0.04–0.40); 0.02	0.43	0.46 (0.08–0.84); 0.02	0.60
	PLAY	1.48 (0.66; 35)	1.23 (0.78; 34)	0.22 (0.02–0.43); 0.04	0.33		
SCL-DBD (CD)	THAV	0.17 (0.14; 48)	0.13 (0.10; 39)	0.03 (– 0.02 to 0.07); 0.26	0.21	0.07 (– 0.01 to 0.16); 0.10	0.41
	PLAY	0.22 (0.17; 35)	0.21 (0.20; 33)	0.01 (– 0.05 to 0.06); 0.87	0.06		
SCL-DBD (prosocial behavior)	THAV	1.92 (0.41; 48)	1.97 (0.40; 42)	– 0.05 (-0.19 to 0.09); 0.45	– 0.12	– 0.03 (– 0.47 to – 0.10); 0.01	– 0.71
	PLAY	1.76 (0.44; 35)	1.66 (0.51; 34)	0.10 (– 0.06 to 0.26); 0.23	0.23		
SCL-ADHD (total)	THAV	0.95 (0.58; 48)	0.80 (0.57; 42)	0.16 (0.01–0.30); 0.04	0.28	0.13 (– 0.14 to 0.40); 0.35	0.22
	PLAY	1.14 (0.59; 35)	0.99 (0.54; 34)	0.12 (– 0.04 to 0.29); 0.15	0.20		
CBCL (internalizing problems)	THAV	0.21 (0.18; 48)	0.18 (0.16; 41)	0.02 (– 0.02 to 0.07); 0.30	0.11	0.07 (– 0.00 to 0.13); 0.06	0.49
	PLAY	0.27 (0.22; 35)	0.19 (0.17; 31)	0.06 (0.00 to 0.11); 0.04	0.27		
CBCL (externalizing problems)	THAV	0.39 (0.20; 48)	0.31 (0.17; 41)	0.06 (– 0.01 to 0.12); 0.08	0.30	0.12 (– 0.01 to 0.25); 0.07	0.45
	PLAY	0.56 (0.26; 35)	0.47 (0.30; 33)	0.06 (– 0.01 to 0.14); 0.09	0.23		

Notes. *FAVK* questionnaire for aggressive behavior*, SCL-DBD* symptom checklist for disruptive behavior disorder, *ODD* oppositional defiant disorder, *CD* conduct disorder, *SCL-ADHD* symptom checklist for attention deficit hyperactivity disorder, *CBCL* child behavior checklist

A comparison of THAV and PLAY at the FU assessment revealed, on a descriptive level, lower problem behavior and higher prosocial behavior in the THAV group compared to PLAY, with effect sizes in the low-to-moderate range (d_THAV/PLAY_ = 0.22–0.71). These contrasts were significant for the scales SCL-DBD *ODD* and SCL-DBD *Prosocial behavior*.

**Fig. 2:**
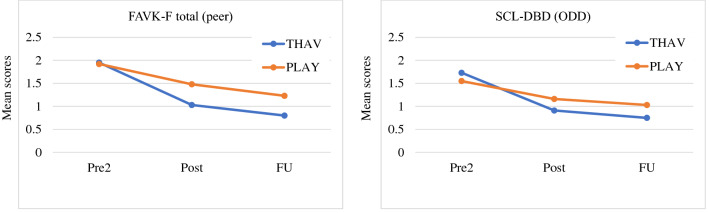
Comparison of mean scores of pre-2-assessment (Pre2), post-assessment (Post), and follow-up assessment (FU) for the FAVK-F total (peer) and the SCL-DBD in THAV and PLAY

Figure 2 illustrates the course of mean values over pre-2 (after the waiting period), post (after the treatment period), and FU (after the FU period) for THAV and PLAY for the primary outcomes (FAVK-F total *Peer* and SCL-DBD *ODD*).

### Clinical significance

The results of the analysis of clinical significance of the changes on the SCL-DBD reveal that compared to pre-treatment, 0.0% of patients in the THAV condition deteriorated, 10% did not change reliably, and 90% improved at FU assessment. In the PLAY condition, 2.5% deteriorated, 39% did not change reliably, and 58.5% improved. A Chi-square test of the distribution of deteriorated or unchanged patients compared to improved patients showed a statistically significant higher percentage of improved patients in the THAV condition (Chi^2^(1) = 32.00, *p* < 0.001).

## Discussion

THAV is an individualized CBT treatment program for boys aged 6–12 years with a diagnosis of ODD/CD, which is combined with parent- (PMT), teacher- and peer-focused interventions. In the previous studies, THAV has been shown to be effective in reducing aggressive behavior problems [11, 22]. In particular, a study employing a within-subject design, which compared the course of symptoms during a 6-week waiting period with the course during the subsequent THAV treatment period, demonstrated a significant decrease in peer-related and adult-related aggressive behavior and a significant increase in prosocial behavior. Furthermore, the RCT evaluation indicated that when compared to an active control involving group play (PLAY), THAV is specifically effective in reducing peer-related aggression, with a low-to-moderate-effect size *d*_THAV/PLAY_ = -0.46) and in improving aggressive and rule-breaking behavior and prosocial behavior, with moderate-effect sizes.

The aim of the present study was to examine the stability of the effectiveness of THAV at a 10-month FU. We hypothesized that the improvements in child behavior, as rated by the parents, through THAV treatment would be sustained 10 months later from post-treatment to FU, and that the significant effects of THAV compared to PLAY shown at post-assessment would also be apparent at the 10-month FU. Overall, the results of our study confirm our hypotheses. Comparable to previous studies on long-term effects of CBT and PMT interventions on ODD/CD [17, 18], the comparison of post-treatment versus 10-month FU indicated the long-term maintenance of treatment outcomes. In particular, in THAV, there was no significant deterioration in symptoms of CD and prosocial behavior, or in problem-maintaining and problem-moderating factors. Moreover, there was even a further significant improvement in oppositional behavior and comorbid ADHD symptoms. Similar to Fossum and colleagues [18], we mostly found small-to-moderate-effect sizes. When clinical significance analysis is considered, it appears that 90% of patients in the THAV condition improved at FU assessment compared to pre-treatment. Similar findings are also seen in the study by Lochman and Wells [13]. Here, it was shown that the Coping Power intervention moved aggression boys’ self-, parent-, and teacher-rated behavior problems from a nonnormative range at pre-treatment to a normative range by the 1-year FU.

The described stability and even partial improvement are evident not only in the THAV but also on almost all scales in the PLAY condition. On the scale of CBCL-*Internalizing behavior*, the PLAY condition even shows a stronger improvement than the THAV condition over the FU period. This can possibly be explained by the fact that the group condition offers good opportunities for improving social skills and an easier transfer to the natural environment. The only exception is the SCL-DBD-*Prosocial Behavior*, which shows a slight, non-significant deterioration in the PLAY condition over the FU time.

When comparing THAV and PLAY at FU, it emerged that the significant differences between the two conditions found at post-treatment assessment were mostly also apparent 10 months later (SCL-DBD-*ODD*, SCL-DBD-*Prosocial Behavior*). In contrast to post-treatment [11], no significant difference between the two conditions was found on the FAVK-F-*Peer* and CBCL-*Externalizing behavior* at FU. However, these values narrowly missed significance (*p* = 0.07). With regard to effect sizes, comparable values between post-treatment and FU were evident, although some values were slightly higher at the FU (SCL-DBD-*CD: d*_post_ = 0.27 *vs. d*_FU_ = 0.41*;* SCL-DBD-*Prosocial behavior: d*_post_ = 0.42 *vs. d*_FU_ = 0.71*; CBCL-Internalizing behavior (d*_post_ = 0.28 *vs. d*_FU_ = 0.49).

To summarize, as the RCT study on post-treatment effects already demonstrated [11], the present findings now indicate that the multimodal approach of THAV is superior to the active group play of PLAY—not only immediately after therapy but also 10 months later. At this point, it is difficult to compare our findings with previous research, because we found only two studies [19, 20] that, besides investigating the stability of effects of CBT (and PMT) on ODD/CD, also looked at the differences between the groups at the FU assessment. The first study, by Cavell and Hughes [19], examined the post-treatment and long-term effects of a CBT program (training in problem-solving skills, and consultation with parents and teachers) compared to a ‘Standard Mentoring’ program (that was carried out with minimally trained, unsupervised mentors) in highly aggressive children. The authors did not find any group differences at the FU assessment. However, the study also did not reveal any significant group differences at the post-treatment assessment. The second study, by Pepler and colleagues [20], investigated the efficacy of a combined CBT and PMT program compared to an untreated comparison group (waiting list) in aggressive girls. The study revealed a difference between the groups at post-treatment, but did not find any significant differences between the groups at FU, with the latter being due to the fact that the control group participants had received adequate therapy in the interim. These two studies are therefore also difficult to compare with our findings, as in our study, there was no significant difference between THAV and PLAY with respect to treatment received during the FU period.

The present study has several strengths and limitations. One strength is that we looked not only at the stability of the THAV treatment but also at the stability of the differences between THAV and PLAY at FU assessment. To the best of our knowledge, our study is the first to demonstrate on one hand that the therapeutic effects of a multimodal therapeutic approach for ODD/CD with a focus on child-based interventions remain stable over a certain period of time, and on the other hand that the effects are superior to those of an active control group in the long term. A further strength of the study is that we examined an active control group instead of a waitlist control group. It can be assumed that the differences found between THAV and the control group would have been even greater if children of the control group had received no therapy at all. Besides other strengths of the study, such as the high percentage of participants (84%) retained in the FU, several limitations have to be mentioned. First, ten patients dropped out from the PLAY condition after randomization because of ethical objections by the therapists. For these patients, the more effective treatment according to the hypotheses of the trial was strictly indicated according to the regulations of the ethics committee, which required that the patients must not be put at a disadvantage due to their participation in the trial. Even though it represents a methodological shortcoming of our study, we decided to exclude the patients from further analyses, since the alternative—an imputation of missing values in this control group (e.g., with last observation carried forward)—would have favored the treatment group and thus the research hypotheses. Second, the results are restricted to boys, and further research should therefore also focus on girls. Third, the influence of callous-unemotional traits was not assessed in the FU. The impact of limited prosocial capacities and of psychopathic or callous-unemotional personalities on the effect of THAV and PLAY should be considered in further studies. Fourth, all outcome measures were based on parent ratings. As such, the results are influenced by the parents' perceptions and expectancies and may be biased due to effort justification. However, this bias may be similar in both conditions. Moreover, parents cannot make an accurate statement about their children’s behavior at school. Here, teacher ratings would have been helpful. However, although we also collect the teachers’ questionnaires at FU assessment, we did not include these in the analyses due to a high number of missing data. Likewise, we also collected self-ratings from the age of 10 years, but here too, there were too few available data. Future studies should consider not only parent ratings but also self- and teacher ratings to draw a more complete picture. Fifth, parents could not be fully blinded, because they actively took part in the intervention. Nevertheless, parents were blinded to the specific hypotheses of the study and many of the parents in the PLAY condition actually believed that this was the intervention that was expected to be more effective. Finally, adherence to the interventions should be considered in further studies. Information on adherence on THAV can be found in the RCT article [11], but an analysis of the differences in adherence between THAV and PLAY also seems interesting, as PLAY may be an effective treatment method and not simply an unspecific control group.

## Conclusion

Overall, the study found that the effects of the individually tailored treatment program THAV, which combines patient-focused (CBT) with parent- (PMT), teacher-, and peer-focused interventions, were retained at a 10-month FU assessment. The effects that were found at the end of the treatment remained stable and even partially improved over the FU period. Additionally, the differences between THAV and the active control group PLAY, which were apparent at the end of the treatment (post), mainly also persisted 10 months later.

## Data Availability

The datasets generated and analyzed during the current study are not publicly available due to obligation to secrecy toward the participants.
